# 

**Published:** 2011-05-25

**Authors:** F Popa

**Affiliations:** ‘Carol Davila’ University of Medicine and PharmacyRomania

Journal of Medicine and Life is actually upgrading, in order to better support the parameters of evaluation that scaffold the correlation of the local research with the international one, which, in turn, is rapidly developing and improving. Accordingly, the journal itself will better follow the international recommendations as far as the manuscripts evaluations are concerned. This means that a stronger network of external reviewers entered in use, to provide a professional evaluation for each paper submitted and to support the decisions of the editorial team. This, in turn, will improve the quality of the papers published in Journal of Medicine and Life and will raise the prognostic of visibility for each and all the studies published in the journal. 

The benefit obtained by a modern editorial management is bilateral. On one hand, the journal will benefit from strong papers that will be of great help in raising the indexing level of the journal. On the other hand, stronger journal indexations will be a better offer for those authors in need of good research indicators to support various funding resources or other criteria, professional and administrative. 

The interdependence loop, journal–to–authors, is complete only if the international criteria for a scientific journal are consistently supported by the authors’ submissions. This is why, we strongly encourage the authors to observe and carefully respect the upgraded Instructions for Authors, which are actually in use. Not complying with this major requirement, means that the submitted manuscripts will be delayed or directly rejected, with or without the chance for a resubmission.

Along with the upgrade of the editorial criteria of selection, a better competition between manuscripts will develop, and, only those scientifically strong and technically well prepared will have the chance to be included in the publication. This is not only a competition between the Romanian authors, but also between authors from various international research media, who will increasingly enter the game. Only the best submission will qualify. 

In accordance with the international trends in research, the journal will pay a special attention to those studies approaching modern topics of large interest, and researches conducted towards various exploratory niches. This will ensure a better entrance on the market of research and funds raising, so, the journal must not be regarded only as an administrative tool which supports promotions.

